# Adverse reaction to benzathine benzylpenicillin due to soy allergy: a case report

**DOI:** 10.1186/s13256-015-0602-z

**Published:** 2015-06-06

**Authors:** Simona Barni, Francesca Mori, Stefano Pantano, Elio Novembre

**Affiliations:** 1 Allergy Unit, A. Meyer Children’s Hospital, Department of Pediatrics, University of Florence, Viale Pieraccini 24, 50139 Florence, Italy; 2 Cystic Fibrosis Department, San Liberatore Hospital, Via Risorgimento, 64032 Atri, Teramo Italy

**Keywords:** Drug allergy, Hidden allergen, Soy lecithin, Soybean allergy

## Abstract

**Introduction:**

Soybean allergy is one of the most common food allergies especially among children. The Food Allergen Labeling and Consumer Protection Act (FALCPA) in the US requires the labeling of soy lecithin because it is derived from soybeans and may contain a number of IgE-binding proteins, possibly representing a source of hidden allergens. Here we describe a pediatric case of soy allergy misunderstood as drug allergy.

**Case presentation:**

An 11-year-old Caucasian girl was referred to our Allergy Unit because of the delayed appearance of an itching papular rash at the site of an injection of benzathine benzylpenicillin delivered by prefilled syringe. A skin test with benzathine benzylpenicillin and detection of serum-specific IgE to penicilloyl V, penicilloyl G, ampicillin and amoxicillin were negative. From her past medical history we know that, at the age of three years, she presented with edema of the lips and difficulty in breathing after eating a soy ice-cream. For that reason, she underwent a skin prick test with soybean that was negative and a serum-specific IgE to soybean test that was weakly positive (0.21KU/L). She underwent an oral provocation test with soy milk that yielded a positive result.

**Conclusions:**

We describe a case of a patient with a delayed reaction to soy as a hidden allergen in a benzathine benzylpenicillin prefilled syringe. This case shows that lecithin contaminated by soy proteins and used as an excipient in drugs can cause reactions in patients with soy allergy. For that reason, the source of lecithin should always be specified among the constituents of drugs to avoid a source of hidden allergens and difficulties in the allergy work-up.

## Introduction

Soybean allergy is one of the most common food allergies especially among children. Approximately 0.4% of children are allergic to soy but about half of them spontaneously recover by the age of 7 years [1].

Patients with soy allergy show a wide range of immunoglobulin E (IgE) and non IgE-mediated clinical symptoms [2]. For these reasons, the Food Allergen Labeling and Consumer Protection Act (FALCPA) in the US, from January 1, 2006, mandates labeling of all ingredients derived from commonly allergenic foods, including soybeans, with the exception of highly refined oils [3]. The FALCPA also requires the labeling of soy lecithin because it is derived from soybeans and may contain a number of IgE-binding proteins, possibly representing a source of hidden allergens [4]. Soy lecithin is not only used in the food industry, as an antioxidant, but it is widely used in topical skin care products, in various drugs administered either topically, orally, intravenously or by inhalation, as an emulsifier [5]. Here we describe a pediatric case of soy allergy misunderstood as drug allergy.

## Case presentation

**Figure 1:**
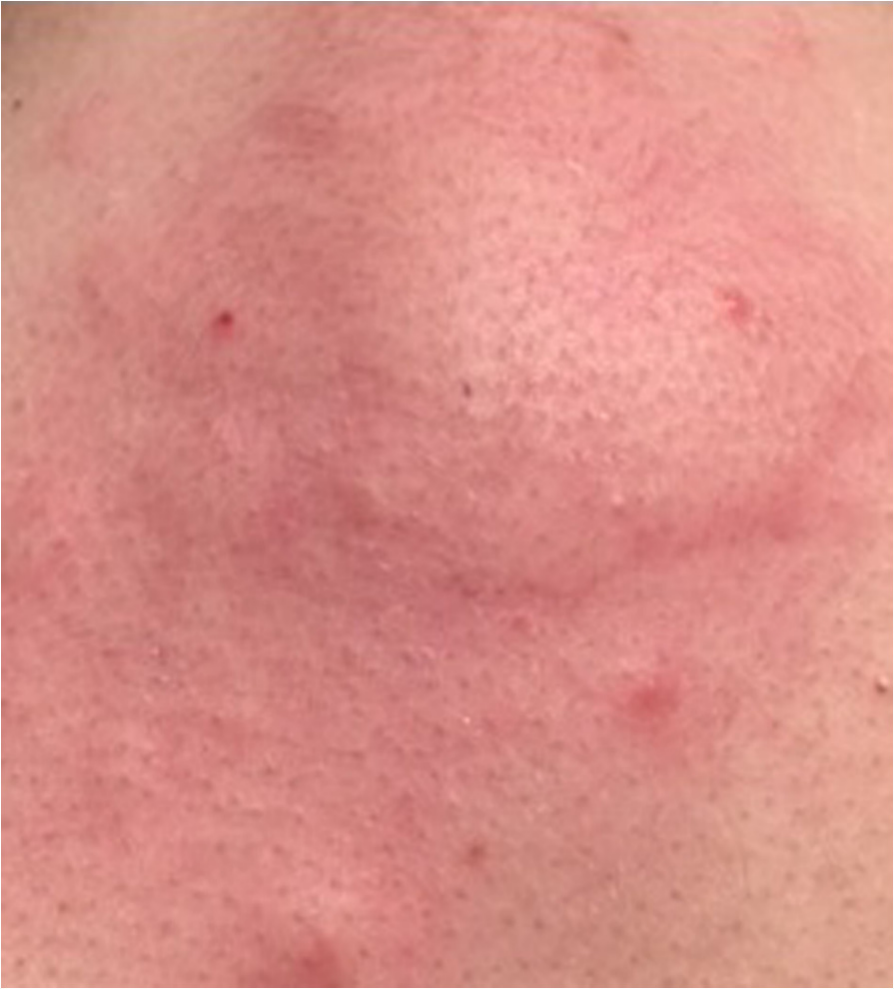
Skin reaction at the site of the benzathine benzylpenicillin injection.

An 11-year-old Caucasian girl was referred to our Allergy Unit because of a delayed appearance (that is after 12 hours) of an itching papular rash at the site of an injection of benzathine benzylpenicillin 1,200,000 UI (Biopharma, Rome, Italy) delivered via prefilled syringe (Figure 1).

From the past medical history, at three years of age, our patient had edema of the lips and difficulty in breathing after eating a soy ice-cream. For that reason, she underwent a blood test analysis to measure the level of serum-specific IgE to milk, egg, soybean, tomato, peanut, whey and fish. Specific IgE to soybean resulted weakly positive (0.21 KUA/l) and she was put on a soybean-free diet.

At nine years of age, because of a post-traumatic spleen breakage, our patient was splenectomized and from that moment she was put on antibiotic prophylaxis with intramuscular benzathine benzylpenicillin (Biopharma) every 21 days. Our patient tolerated 14 injections of the drug in the formulation powder and using solvent for reconstitution. On January 2012, because of the exit from the market of this formulation, a new formulation, packaged as a prefilled syringe, was used. After the first use, our patient presented with a delayed papular rash at the injection site, as mentioned above.

A skin prick test and intradermal test with benzylpenicillin at a concentration of 10,000 UI and a patch test at 5% full-strength concentration were all negative. Moreover, the detection of serum-specific IgE to penicilloyl V, penicilloyl G, ampicillin and amoxicillin was negative; a skin prick test with soybean was negative and a serum-specific IgE to soybean was weakly positive (0.21KUA/l). Three months after the reaction mentioned above, and after receiving informed consent from the girl and her parents, we decided to perform an oral provocation test (OPT) with cefpodoxime proxetil (200mg) that yielded a negative result. We also performed an OPT with soymilk (50ml divided into three doses) with no reaction after two hours of observation on the first day; however, on the second day, she presented with diarrhea and abdominal pain. We provide a summary of our patient’s clinical findings (Table 1).

**Table 1:** **Clinical findings**

Race	Caucasian
Sex	female
Age	11 yrs
Respiratory allergy	none
Food allergy	soy
Past medical history	splenectomized after an accident injury

Taking into account the following factors: (a) the presence of soy lecithin among the constituents of benzathine benzylpenicillin (Biopharma) both in the prefilled syringe and in the formulation powder and solvent for reconstitution; and (b) the positive medical history for soy allergy, a cause and effect link between soy lecithin and the allergic reaction was suspected. Moreover, the positive OPT with soymilk reinforced the hypothesis of a delayed allergy to soy proteins contaminating the lecithin contained in the prefilled syringe.

According to the current guidelines [6], we decided to change the antibiotic prophylaxis to orally administered cefpodoxime proxetil every 21 days and she had no further reactions.

## Discussion

We describe a case of a patient with a delayed reaction to soy as an hidden allergen in a benzathine benzylpenicillin prefilled syringe.

Allergic reactions to soy lecithin have been previously reported in the literature: occupational asthma in bakers [7], chronic diarrhea in a child [8], and anaphylactic reaction in a 40-year-old woman treated with an inhalatory drug [9].

We hypothesized that beside the history of immediate IgE-mediated systemic reaction to soy, the skin reaction under investigation was not due to an IgE-mediated mechanism, considering the low level of specific IgE to soy and the delayed onset of her symptoms, but most likely it could be due to a cell-mediated reaction, as confirmed by the OPT.

Gu *et al*. [4] demonstrated that soy lecithin contains a number of IgE-binding proteins thus it might represent a source of hidden allergens. The presence of these allergens is a most significant concern for soy-allergic individuals consuming lecithin products as a health supplement. Presently, lecithin products used as health supplements are not labeled as containing soy protein. Although both formulations contain soy lecithin, we hypothesized that the reasons why our patient only reacted to the prefilled syringe were: (a) a different purification of lecithin extracted from soy, and (b) a progressive sensitization to soy proteins over the course of the antibiotic therapy.

## Conclusions

This case shows that lecithin contaminated by soy proteins and used as an excipient in drugs can cause reactions in patients with soy allergy. For that reason, the source of lecithin should always be specified among the constituents of drugs and on the labels of food products to avoid a source of hidden allergens and difficulties in the allergy work-up.

## Consent

Written informed consent was obtained from the patient’s legal guardian for publication of this case report and any accompanying images. A copy of the written consent is available for review by the Editor-in-Chief of this journal.

## Abbreviations

FALCPA: Food Allergen Labeling and Consumer Protection Act
IgE: immunoglobulin E
OPT: oral provocation test
